# Atypical Manifestation of Skull Base Osteomyelitis Secondary to Malignant Otitis Externa Following Radiotherapy: A Case Report

**DOI:** 10.7759/cureus.92874

**Published:** 2025-09-21

**Authors:** Mohamed Bouallou, Zakaria Essamhi, Achraf Sbai, Drissia Benfadil, Azzedine Lachkar, Fahd El Ayoubi El Idrissi

**Affiliations:** 1 Department of Otolaryngology - Head and Neck Surgery, University Hospital Centre Mohammed VI, Oujda, MAR

**Keywords:** ciprofloxacin, malignant otitis externa, otorrhagia, pseudomonas aeruginosa, radiotherapy (rt), skull base osteomyelitis

## Abstract

Skull base osteomyelitis represents a rare but potentially fatal complication of malignant otitis externa (MOE), predominantly affecting elderly patients with diabetes or immunosuppression, and may also occur in individuals with a history of head and neck radiotherapy.

We report the case of a 79-year-old female with poorly controlled diabetes and a remote history of undifferentiated carcinoma of the nasopharynx (UCNT) treated with radiotherapy 17 years prior, who neglected chronic, fluctuating otalgia. She presented with massive left-sided otorrhagia complicated by hemorrhagic shock and a left peripheral facial palsy graded House-Brackmann IV. High-resolution imaging demonstrated extensive destruction of the temporal bone and skull base, and microbiological cultures identified *Pseudomonas aeruginosa*. Prompt, prolonged targeted antibiotics led to complete clinical recovery. This case underscores the critical importance of early recognition, meticulous longitudinal follow-up of post-radiotherapy patients, and aggressive multidisciplinary management to prevent potentially life-threatening complications. The unusual presentation of this case makes it worthy of sharing.

## Introduction

Skull base osteomyelitis represents a serious complication of malignant otitis externa (MOE). This condition predominantly affects elderly diabetic patients, likely attributable to the elevated cerumen pH and microangiopathic changes of the external auditory canal observed in this population [1].

Although malignant external otitis does not represent a neoplastic entity, the designation 'malignant' was coined by Chandler in 1968 to characterize its aggressive progression and destructive potential [2]. The causative agents are usually bacteria and fungi. In more than 90% of cases, *Pseudomonas aeruginosa* serves as the causative pathogen. Temporal bone osteomyelitis may result in cranial nerve palsies once the infection extends through the fissures of Santorini and the tympanomastoid suture, infiltrates the Haversian system of the compact bone, and ultimately reaches the skull base [3]. Owing to its anatomical proximity to the external auditory canal upon exiting the skull base, cranial nerve VII is most frequently involved, followed sequentially by cranial nerves IX, X, XI, and XII [4]. Initially, the predominant symptoms include otalgia and headache, often accompanied by ear discharge and conductive hearing loss, which typically result from external auditory canal swelling or the formation of granulation tissue [5]. However, otorrhagia is rarely reported in the literature. At this stage, computed tomography (CT) may identify fluid within the mastoid air cells, while magnetic resonance imaging (MRI) delineates inflammatory involvement of the skull base structures. Management of skull base osteomyelitis secondary to malignant otitis externa requires prolonged antibiotic therapy, often complemented by surgical debridement, with antifungal agents reserved for selected cases [6].

## Case presentation

We report the case of a 79-year-old female with multiple comorbidities, including poorly controlled type 2 diabetes mellitus, hypertension, ischemic heart disease, and a history of left forearm fracture managed with osteosynthesis five months earlier. The patient also had a history of undifferentiated carcinoma of the nasopharynx (UCNT) treated with radiotherapy and declared in remission for the past 17 years. The patient reported chronic, fluctuating otalgia, largely disregarded, without other associated symptoms. Furthermore, the patient had no recent history of cranial trauma and no prior history of otologic surgical intervention.

She was transferred to our Department of Otolaryngology-Head and Neck Surgery after a 10-day stay in the intensive care unit, where she had been stabilized for hemorrhagic shock secondary to cataclysmic left-sided otorrhagia.

During hospitalization, local hemostatic measures were undertaken, consisting of repeated Pop-Otowick packing combined with Surgicel, changed daily. The bleeding episodes recurred particularly during hypertensive peaks, despite otherwise stable hemodynamic parameters.

**Figure 1:**
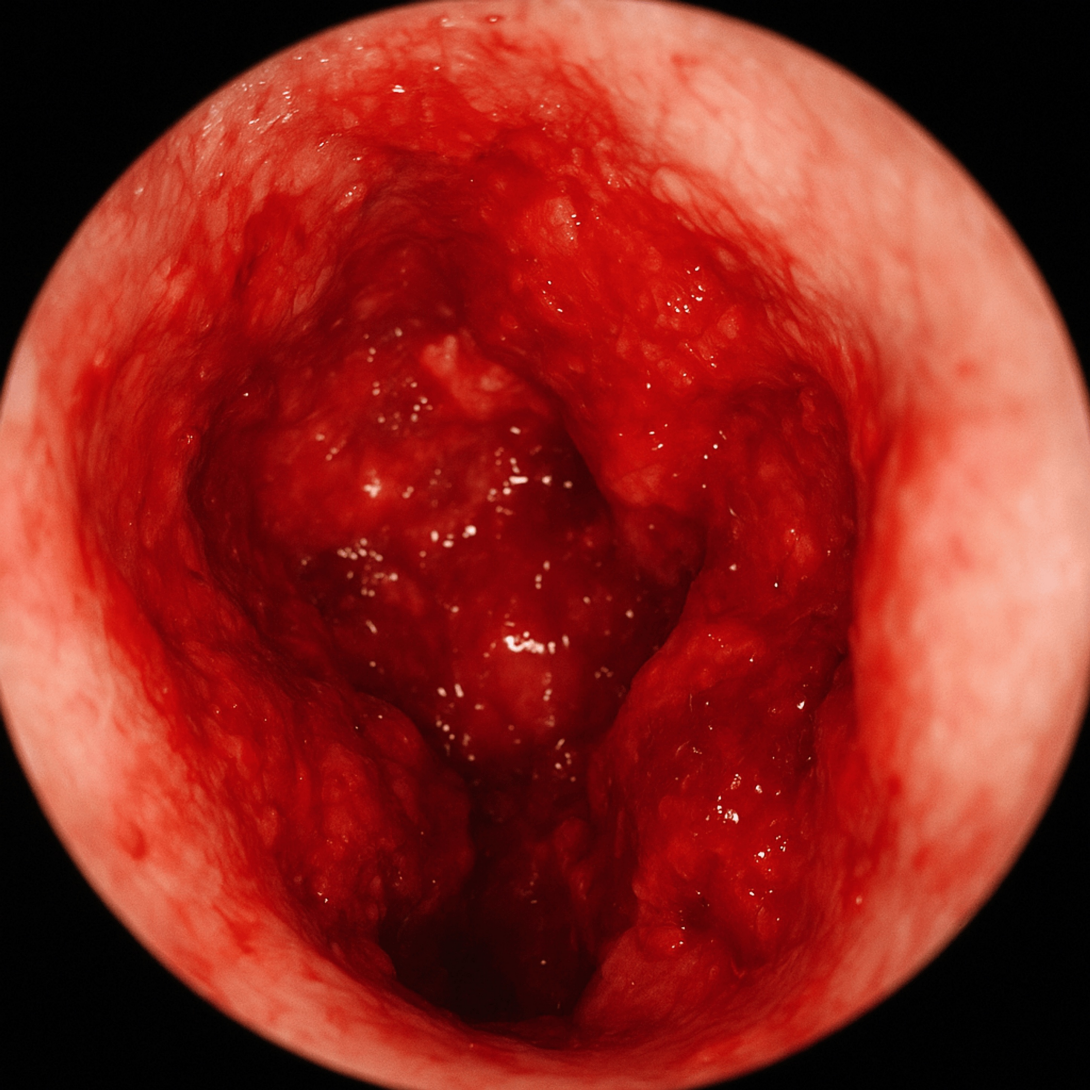
Otoscopic view of the left ear showing a markedly hemorrhagic external auditory canal with diffuse active bleeding obscuring the tympanic membrane, and edematous, irregular canal walls.

**Figure 2:**
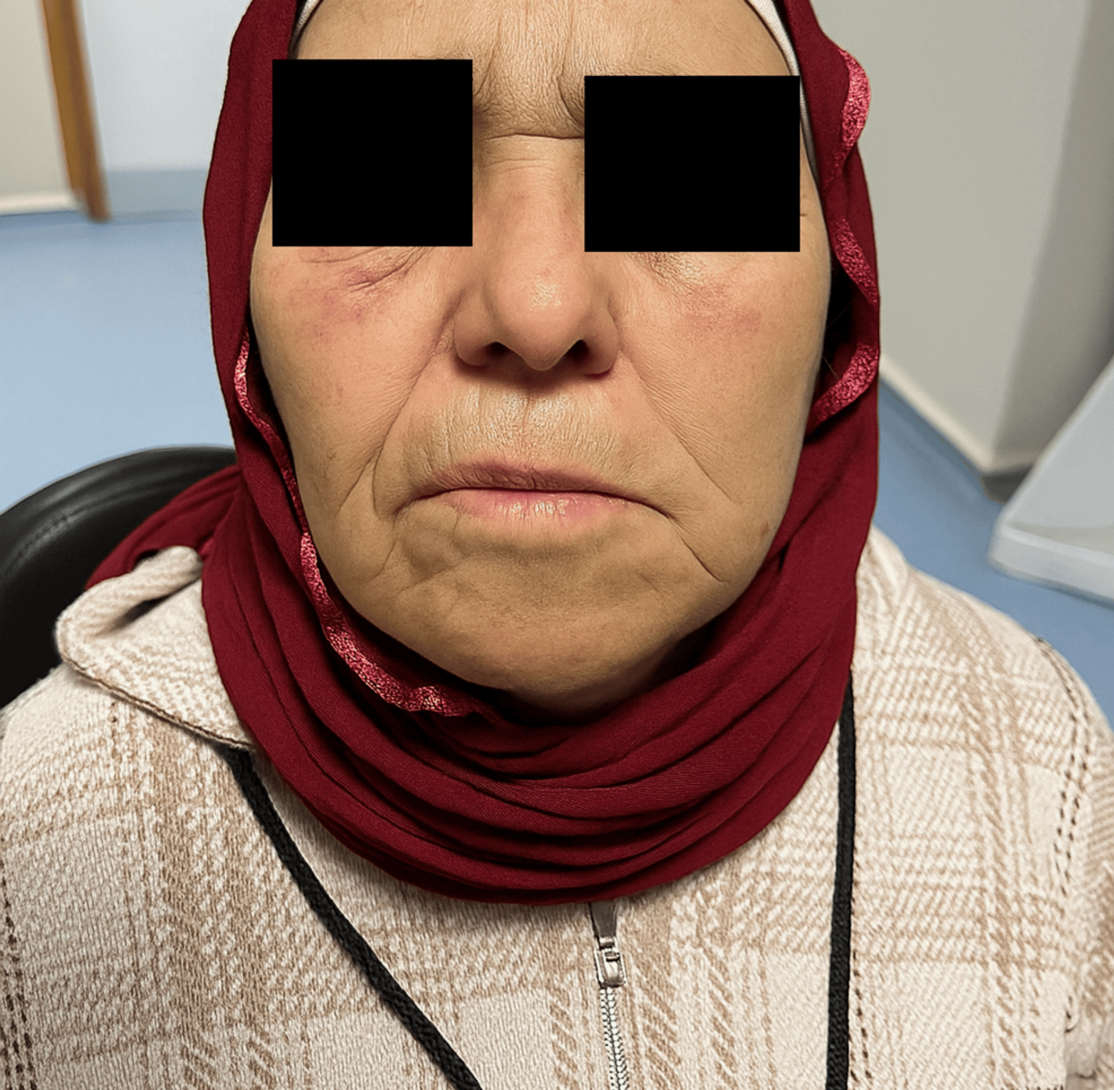
Image demonstrating left-sided peripheral facial paralysis, graded as House–Brackmann IV.

On admission, general examination revealed a conscious patient (Glasgow Coma Scale score 15/15), afebrile (37.1°C), normotensive (120/71 mmHg), and with normal oxygen saturation (99%). Otoscopic evaluation of the left ear demonstrated mild stenosis of the external auditory canal, associated with blood residues and ongoing hemorrhagic oozing, which impeded visualization of the tympanic membrane (Figure 1). Otoscopic examination of the right ear revealed a normal-appearing external auditory canal and an intact tympanic membrane. The vestibular examination revealed no abnormalities. Neurological assessment demonstrated a left peripheral facial paralysis graded House-Brackmann IV, with no additional motor deficits (Figure 2). The remainder of the clinical assessment demonstrated no noteworthy findings.

Pure-tone audiometry performed on day 21 of hospitalization demonstrated a conductive hearing loss, with a threshold reduction of 53 dB in the left ear and 41 dB in the right ear.

**Figure 3:**
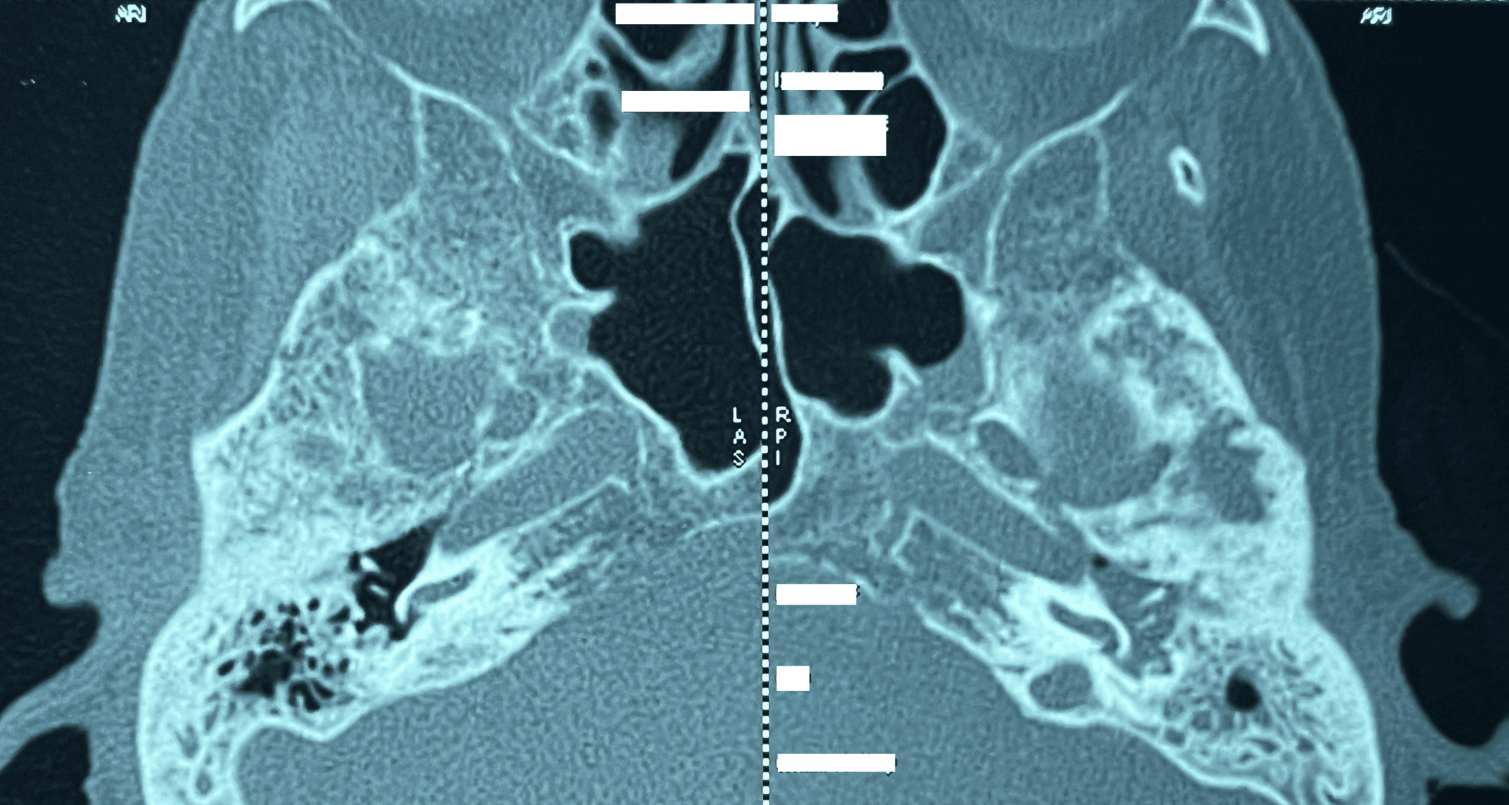
Axial CT image in the bone window demonstrating bilateral erosion of the temporal bones.

**Figure 4:**
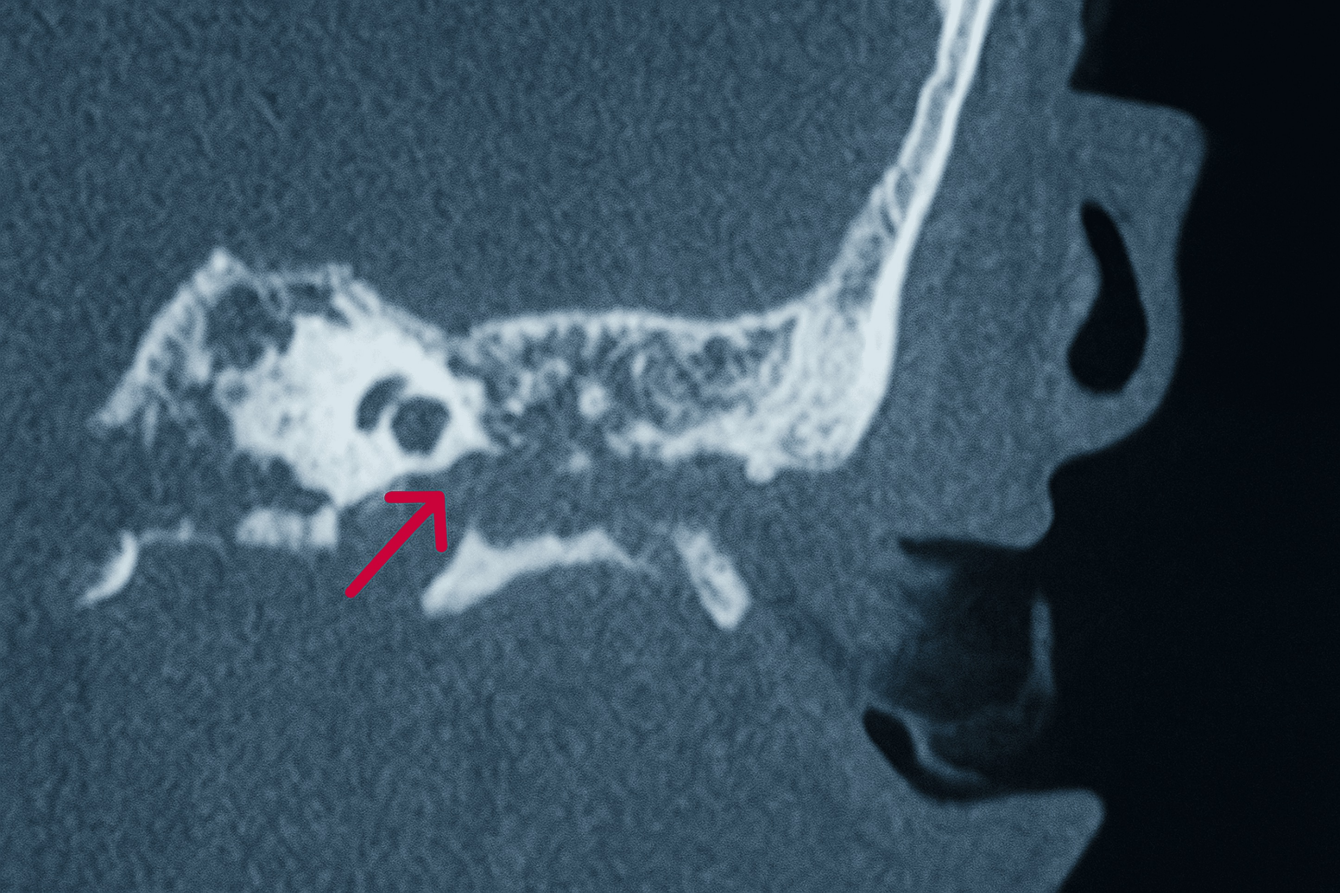
Coronal CT image in the bone window demonstrating lysis in the inferior wall of the left tympanic cavity (red arrow).

High-resolution CT of the temporal bone demonstrated, on the left side, complete opacification of the external auditory canal, middle ear, and mastoid air cells. There was evidence of heterogeneous bone destruction involving the tympanic bone with extension to the skull base, osteolysis of the mandibular condyle associated with temporomandibular joint subluxation, and lytic changes affecting the walls of both the external and internal auditory canals as well as the inferior wall of the tympanic cavity (Figure 3). On the right side, the temporal bone exhibited changes consistent with post-radiation osteonecrosis. The observed otorrhagia was attributed to bony erosion of the inferior wall of the tympanic cavity, resulting in exposure of the jugular bulb (Figure 4). Moreover, no evidence of abscess formation was identified on the CT scan. MRI was not performed due to the presence of ferromagnetic material (osteosynthesis plate).

Following a multidisciplinary meeting with radiologists, the diagnosis of malignant otitis externa complicated by skull base osteomyelitis was established.

At admission, laboratory investigations revealed a white blood cell count of 17,050/µL, platelet count of 221,000/µL, prothrombin time of 100%, and C-reactive protein (CRP) level of 123 mg/L.

Microbiological culture of ear swabs isolated* Pseudomonas aeruginosa*, which was found to be sensitive to ciprofloxacin, with no fungal pathogens identified and negative results for *Mycobacterium tuberculosis*.

The patient has a 47-year history of diabetes mellitus, managed with insulin, with poor therapeutic adherence. Glycated hemoglobin (HbA1c) at admission was 8.1%. The patient received diabetes education, with insulin dose adjustments made in concertation with endocrinologists to normalize blood glucose levels.

**Figure 5:**
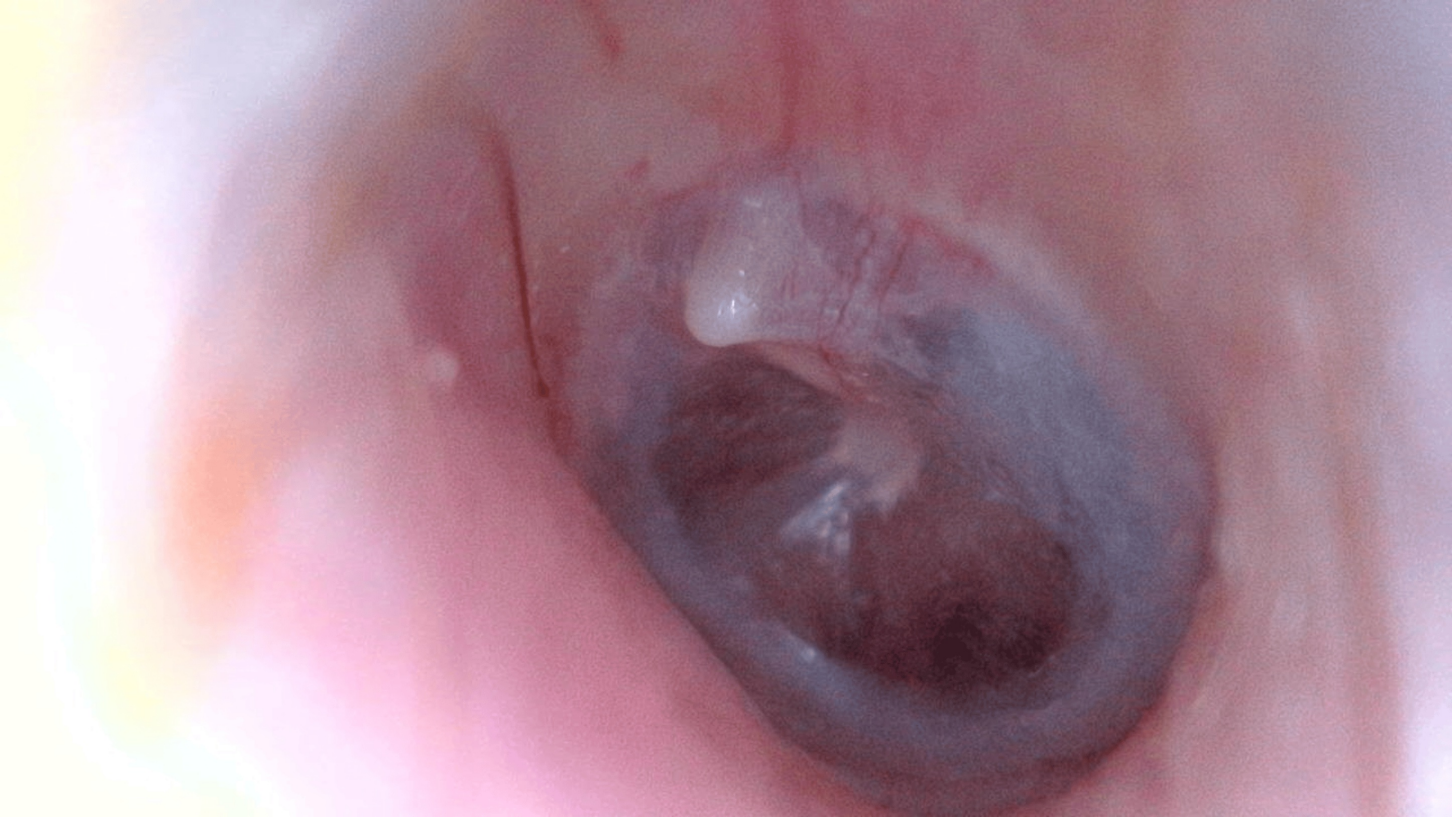
Follow-up otoscopy at day 121 demonstrated marked resolution of local inflammatory signs and complete cessation of otorrhagia.

**Figure 6:**
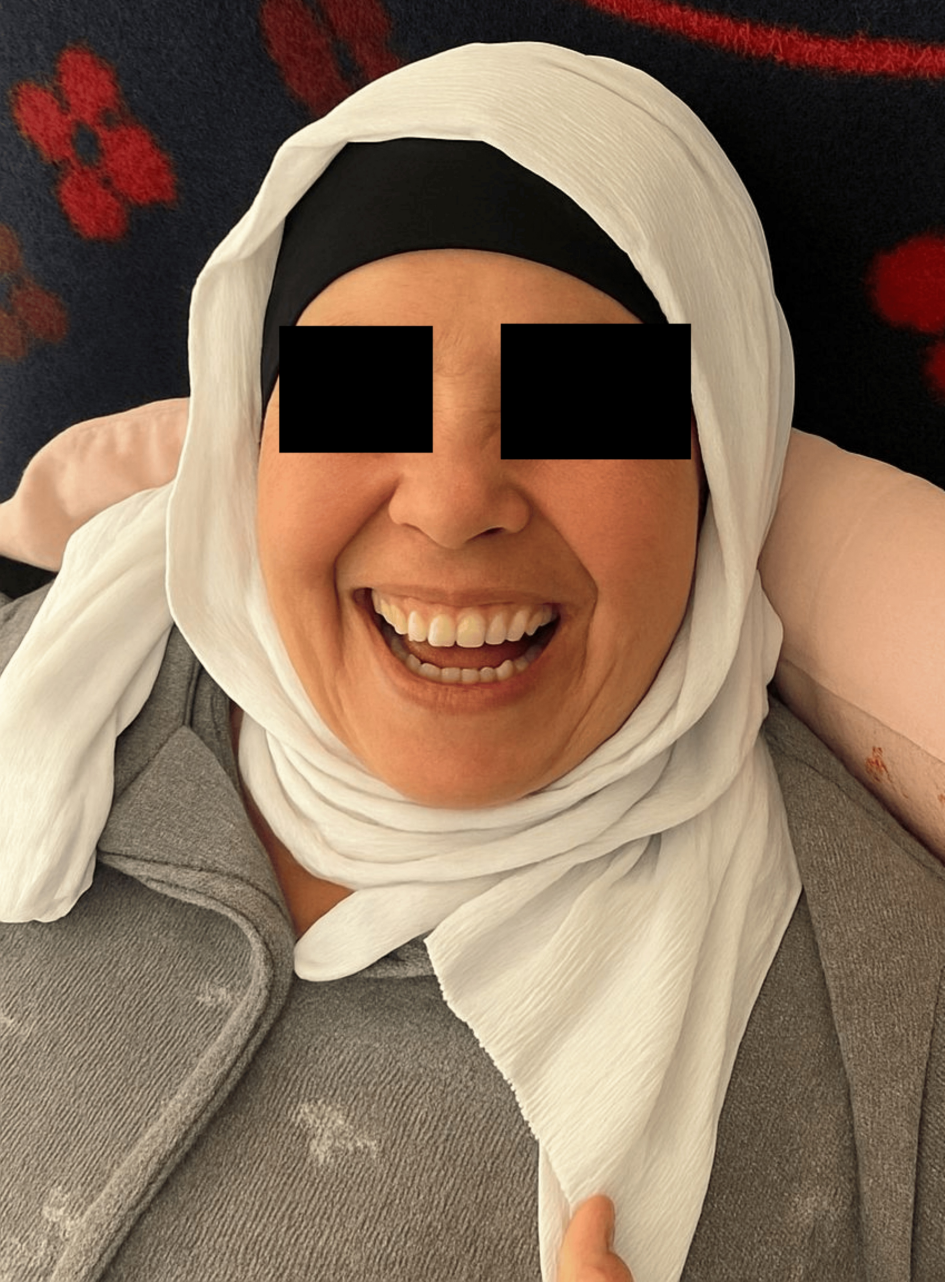
Image demonstrating regression of facial paralysis (Day 80).

Targeted antimicrobial therapy was initiated, consisting of intravenous ciprofloxacin (800 mg/day) in combination with ceftriaxone (2 g/day), supplemented with corticosteroids: dexaméthasone (1 mg/kg) administered for one week with gradual tapering, as well as analgesics and concurrent facial physiotherapy. After optimal management of hypertension and diabetes mellitus with insulin therapy and dietary modifications, the multidisciplinary treatment resulted in significant clinical and laboratory improvements (CRP: 13 mg/L), including resolution of inflammation in the external auditory canal (Figure 5) and regression of facial paralysis (Figure 6). The patient was discharged on the 58th hospital day, and therapy was transitioned to oral ciprofloxacin (800 mg/day) for a duration of 12 weeks. Otologic management included topical ciprofloxacin therapy administered three times per week. Pop Otowick dressings were replaced every five days, and arterial blood pressure was regularly monitored during the six-month follow-up period.

## Discussion

Malignant otitis externa is a severe, progressive infection of the temporal bone that predominantly affects elderly diabetic or immunocompromised patients. In certain cases, the infection may extend beyond the temporal bone to involve the skull base, resulting in skull base osteomyelitis, a potentially life-threatening complication [7]. Extension of the infectious process to the stylomastoid foramen can precipitate facial nerve palsy [8]. Furthermore, parotitis and trismus may ensue secondary to masseter myositis and involvement of the temporomandibular joint.

Microangiopathy and impaired local blood flow in diabetic patients, along with prior radiotherapy, are considered key contributors to the pathogenesis of malignant otitis externa [9]. In the external ear, late radiotherapy-induced reactions may manifest months after treatment as acute or chronic otitis externa, cutaneous ulceration, and necrosis of the osseous or cartilaginous structures [9]. Moreover, radiotherapy-related otologic complications have also been documented, including tympanic membrane sclerosis and perforation [10]. Both risk factors were present in our patient. It is plausible that prior radiotherapy initiated a protracted process of temporal bone necrosis, which, over 17 years, rendered the tissue susceptible to secondary bacterial colonization and subsequent infection, a process further facilitated by the patient’s underlying diabetes mellitus.

The infection typically originates as external otitis, manifesting with otalgia, otorrhea, and aural fullness, and may progressively extend to the middle ear and skull base, conferring substantial morbidity and mortality due to the anatomical proximity of critical structures [11]. Extension into the temporal bone occurs via the fissures of Santorini and the tympanomastoid suture, with subsequent involvement of the stylomastoid and jugular foramina [12]. Venous channels and fascial planes further facilitate dissemination along the dural venous sinuses, ultimately reaching the petrous apex. This pathophysiological progression can result in severe and potentially fatal complications, including mastoiditis, skull base osteomyelitis, cranial neuropathies, cerebral venous sinus thrombosis, arteritis and/or intracranial arterial pseudoaneurysms, and intracerebral abscess formation [11].

Malignant otitis externa should be strongly suspected in any diabetic or immunocompromised patient presenting with otitis externa, given the high risk of progression to skull base osteomyelitis [13]. Clinical manifestations typically encompass severe otalgia, purulent otorrhea, and, in some cases, involvement of the cranial nerves. Owing to its anatomical proximity to the external auditory canal upon exiting the skull base, cranial nerve VII is most frequently involved, followed sequentially by cranial nerves IX, X, XI, and XII [4]. However, otorrhagia has not been previously reported as a presenting symptom. Moreover, extension of the infectious process to the cervical spine may occur in the context of skull base osteomyelitis [14]. Malignant external otitis is initially confined to the soft tissues of the external auditory canal; however, when the infectious process extends to involve the temporal bone or adjacent skull base structures, it is more appropriately designated as skull base osteomyelitis [15]. In our case, the patient’s history of radiotherapy likely compromised the integrity of the temporal bone, thereby facilitating the extension of infection to the skull base.

*Pseudomonas aeruginosa *remains the predominant pathogen implicated in the development of osteomyelitis secondary to malignant otitis externa, accounting for the vast majority of reported cases [16]. Moreover, the involvement of other pathogens has been documented, including *Aspergillus* species, Gram-positive cocci, *Mycobacterium *species, and *Candida *[17]. This rationale underlies the need for prolonged antimicrobial therapy in the treatment of skull base osteomyelitis, emphasizing the critical role of bacteriological cultures in guiding antibiotic selection and optimizing antibiotic efficacy. Fortunately, in our case, the *Pseudomonas aeruginosa* isolate was sensitive to ciprofloxacin.

A thorough diagnostic workup, incorporating advanced imaging modalities and complementary investigations, is essential for accurately delineating the extent of the infectious process and identifying potential complications. Radiological assessment represents a pivotal component in the diagnostic workup of skull base osteomyelitis, not only enabling early detection and precise delineation of the infectious process, but also facilitating the identification of associated complications such as intracranial extension, vascular involvement, and cranial nerve compression. CT is typically employed as an initial imaging modality in the diagnostic evaluation of suspected skull base osteomyelitis, allowing for the assessment of soft tissue involvement as well as early osseous erosion and cortical demineralization [8]. However, its utility in monitoring therapeutic response remains limited, as the initial demineralization changes often persist despite clinical and microbiological resolution [13]. In our patient, computed tomography revealed that the massive otorrhagia was attributable to erosion of the inferior wall of the tympanic cavity, resulting in exposure of the jugular bulb within the middle ear. Moreover, no evidence of abscess formation was identified on the CT scan. MRI has limited sensitivity for detecting osseous alterations; however, it surpasses CT in delineating soft tissue involvement [8]. In particular, diffusion-weighted magnetic resonance imaging (DW-MRI) offers superior anatomical resolution compared with conventional MRI sequences, enhancing the detection of subtle soft tissue and perineural involvement. In our patient, MRI was not performed due to the presence of ferromagnetic material, which constituted a contraindication. The complementary use of both CT and MRI therefore provides a comprehensive evaluation, guiding both diagnosis and longitudinal management of the disease.

Management of skull base osteomyelitis necessitates a multidisciplinary approach, combining prolonged targeted antimicrobial therapy with supportive measures and, in select cases, adjunctive surgical. 

The patient was managed exclusively with antibiotic therapy. Surgical intervention, including biopsies of the mastoid cortex and tympanic cavity for histopathological and microbiological evaluation, was proposed; however, the patient declined the procedure. The advent of newer, more effective, and less toxic antimicrobial agents has significantly diminished the role of surgical intervention in managing skull base osteomyelitis. Systemic antipseudomonal antibiotics constitute the cornerstone of therapy for skull base osteomyelitis secondary to malignant external otitis [1]. Fluoroquinolones, particularly ciprofloxacin, are frequently employed in the treatment of malignant external otitis due to their potent activity against *Pseudomonas aeruginosa*, favorable safety profile, and excellent bone penetration [13]. According to previous studies, the majority of patients require a minimum duration of six to 12 weeks of antimicrobial therapy to achieve effective control of the infection [18]. Accordingly, in our case, quinolone associated with third-generation cephalosporin was administered for a total of eight weeks, resulting in complete clinical remission of otalgia and normalization of C-reactive protein, indicative of an effective biological response. Conversely, numerous reports indicate that over 30% of *Pseudomonas aeruginosa* isolates in cases of malignant external otitis exhibit resistance to ciprofloxacin, likely attributable to its widespread use in the treatment of respiratory infections and as a topical agent for otitis media and externa [19]. This underscores the critical importance of performing bacteriological cultures and antibiotic susceptibility testing on clinical specimens to guide targeted therapy. Indeed, surgical intervention assumes a pivotal role, particularly in instances where conservative management proves inadequate or when there is extensive involvement of the affected tissues.

## Conclusions

Skull base osteomyelitis secondary to malignant otitis externa represents a rare but potentially life-threatening complication, particularly in elderly patients with diabetes and a history of head and neck radiotherapy.

This case illustrates that chronic, neglected otalgia in such high-risk patients may precede catastrophic events, including massive otorrhagia, and involvement of the intracranial vessels - a rare but potentially fatal complication infrequently reported in the literature. Rigorous long-term surveillance of post-radiotherapy patients, coupled with coordinated multidisciplinary management, is essential to mitigate delayed otologic complications, prevent life-threatening sequelae, and optimize both clinical and functional outcomes.
